# Supplementary material for: Temperature alters the predator-prey size relationships and size-selectivity of Southern Ocean fish
Source: Nat Commun. 2024 May 10;15:3979. doi: 10.1038/s41467-024-48279-0 (PMC11087476; doi:10.1038/s41467-024-48279-0)
Supplement: Supplementary file 1 — Supplementary Information [file 41467_2024_48279_MOESM1_ESM.pdf]

Supplementary Information: Temperature alters the size selectivity of Southern Ocean fish

Patrick Eskuche-Keith[1,2], Simeon L. Hill[2], Lucía López-López[3], Benjamin Rosenbaum[4,5], Ryan A. Saunders[2], Geraint A. Tarling[2], Eoin J. O'Gorman[1]

[1] School of Life Sciences, University of Essex

[2] British Antarctic Survey, High Cross, Madingley Rd, Cambridge, CV3 0ET, UK

[3] Ecosystem Oceanography Group GRECO, Oceanographic Centre of Santander (CN IEO, CSIC)

[4] EcoNetLab, German Centre for Integrative Biodiversity Research (iDiv), Halle-Jena-Leipzig, Germany

[5] Institute of Biodiversity, Friedrich Schiller University Jena, Jena, Germany

Supplementary Figures

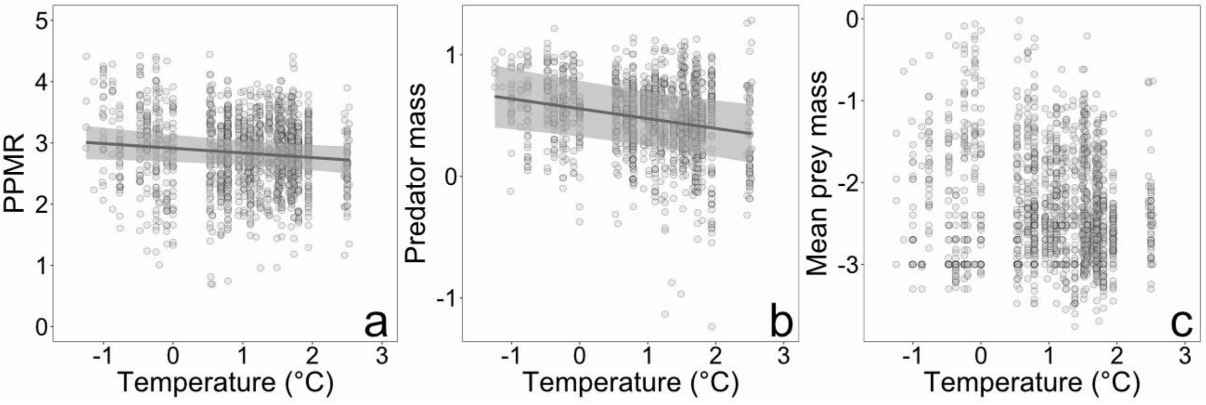

Figure S1: Effects of temperature at 1,062 m depth on predator and prey body mass (*n* = 1576 fish). (a) partial residual plot from a linear mixed model of the effect of temperature (at 1,062m depth) on prey-averaged predator-prey mass ratio (PPMR); (b) partial residual plot from a linear mixed model of the effect of temperature on predator body mass; (c) scatterplot of the relationship between temperature and abundance-weighted average prey mass in predator stomachs. Y-axis values are in $\log_{10}$ g. Lines represent predicted values at each SST. Shading represents 95% confidence intervals.

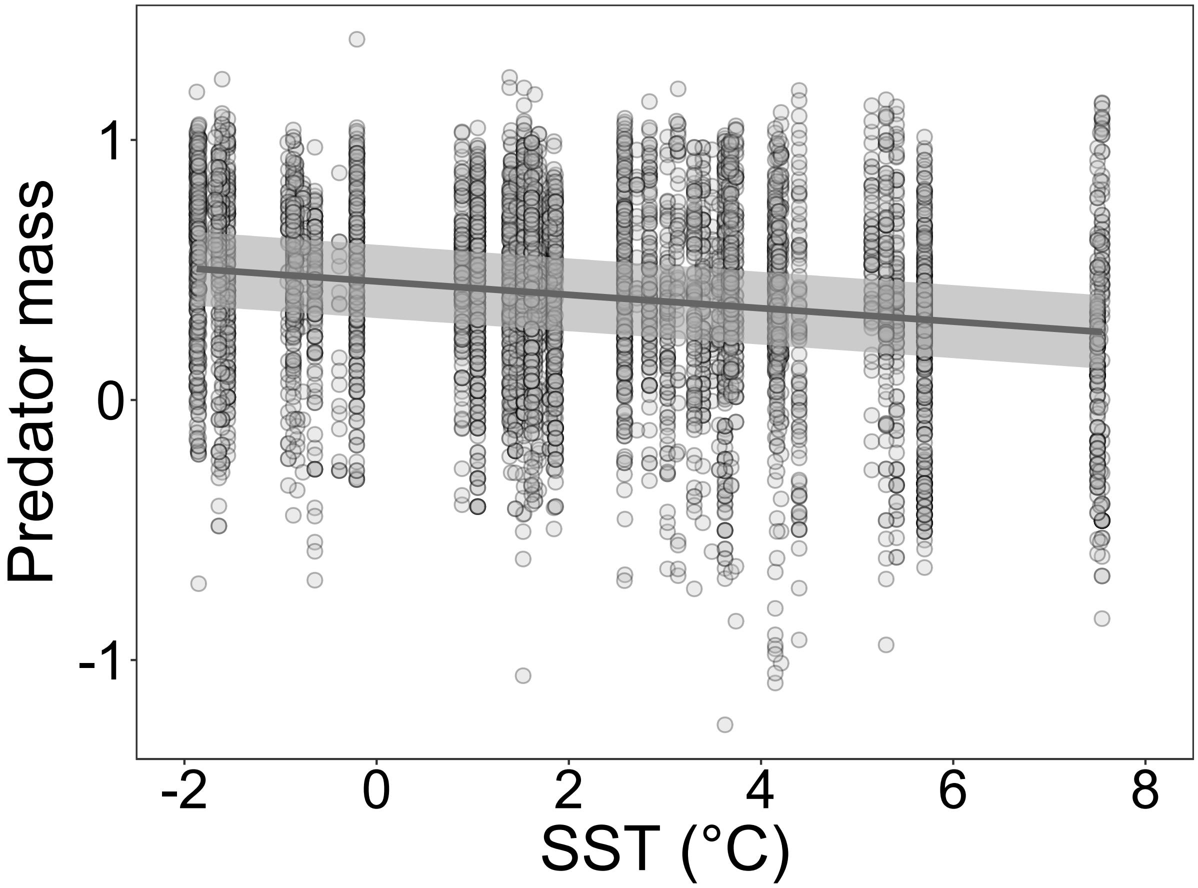

Fig S2: Effects of temperature on predator body mass from a larger dataset. Partial residual plot from a linear mixed model of the effect of sea-surface temperature (SST) on predator body mass using a larger dataset of myctophid body sizes (n = 6,143). Y-axis values are in log_{10} g. Line represents predicted values of predator mass at each temperature. Shading represents 95% confidence intervals.

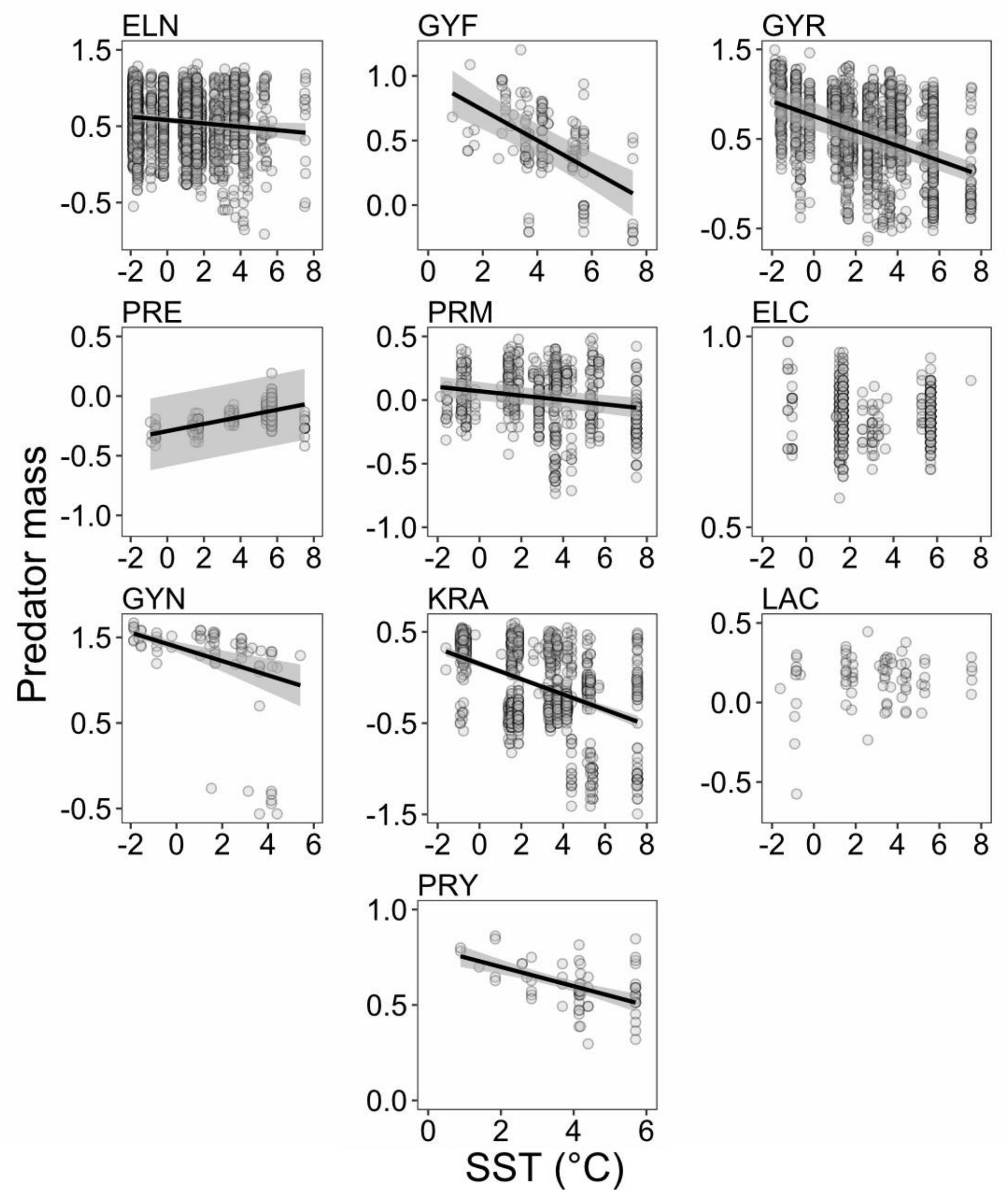

Figure S3: Relationship between predator mass and sea surface temperature (SST) for each species. Panels ELN, GYF, GYR, PRE and PRM are partial residuals plots, the remainder are scatterplots of the raw data with regression lines indicating predicted values from a Generalised Least Squares model. Y-axis values are in $\log_{10}$ g. Panels with no regression lines indicate species for which no significant trend in size with SST was identified. Shading represents 95% confidence intervals. ELC = *E. carlsbergi* (*n* = 486 fish), ELN = *E. antarctica* (*n* = 2,101), GYF = *G. fraseri* (*n* = 143), GYN = *G. nicholsi* (*n* = 68), GYR = *G. braueri* (*n* = 1448), KRA = *K. anderssoni* (*n* = 944), LAC = *N. achirus* (*n* = 76), PRE = *P. tension* (*n* = 217), PRM = *P. bolini* (*n* = 596), PRY = *P. choriodon* (*n* = 64).

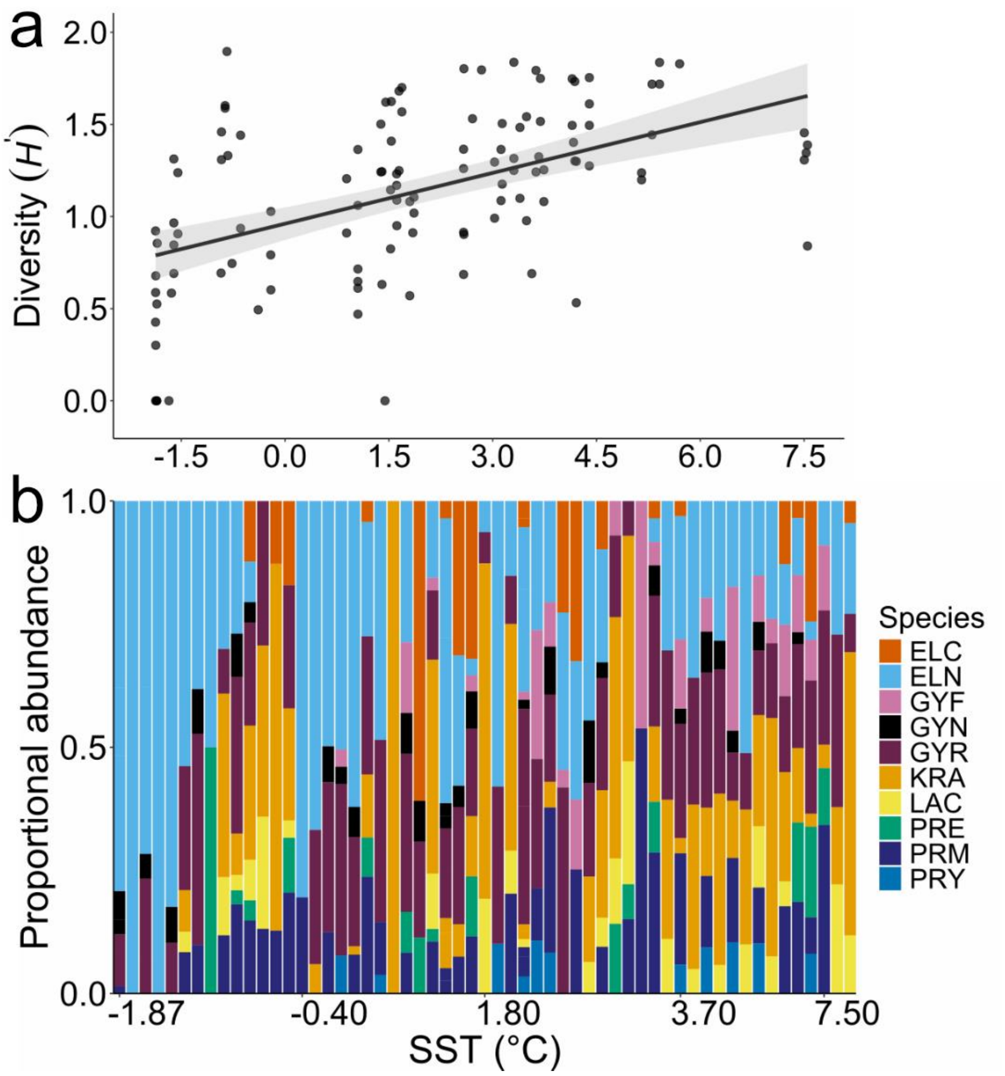

Figure S4: Effects of temperature on myctophid community diversity and the relative abundance of each species. a) Generalised Least Squares regression model predicted values of species diversity (Shannon-Wiener index) versus sea-surface temperature (SST). Shading represents 95% confidence interval. b) stacked bar plot showing the change in proportional sqrt-transformed abundance of each species across SST. Species codes are ELC = *E. carlsbergi* (*n* = 26 individual abundance estimates), ELN = *E. antarctica* (*n* = 101), GYF = *G. fraseri* (*n* = 27), GYN = *G. nicholsi* (*n* = 36), GYR = *G. braueri* (*n* = 91), KRA = *K. anderssoni* (*n* = 64), LAC = *N. achirus* (*n* = 32), PRE = *P. tension* (*n* = 16), PRM = *P. bolini* (*n* = 52), PRY = *P. choriodon* (*n* = 15). A clear shift in species composition can be seen with increasing temperature, from communities dominated by the relatively large-bodied *E. antarctica* at low temperatures to ones with a greater proportion of smaller species like *K. anderssoni* under warmer conditions. Note the discrete x-axis scale for panel b.

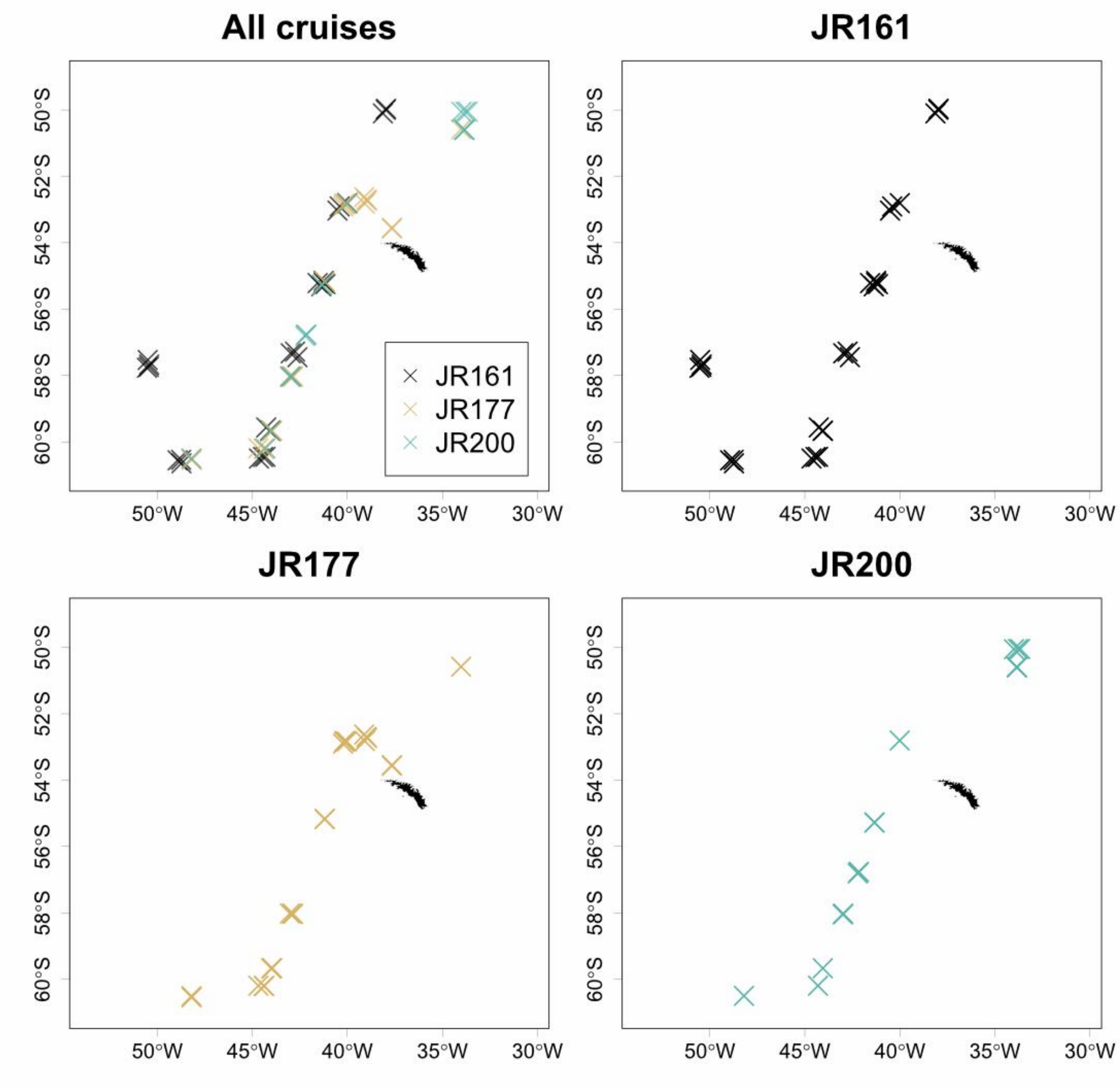

Figure S5: Distribution of myctophid sampling stations from each cruise.

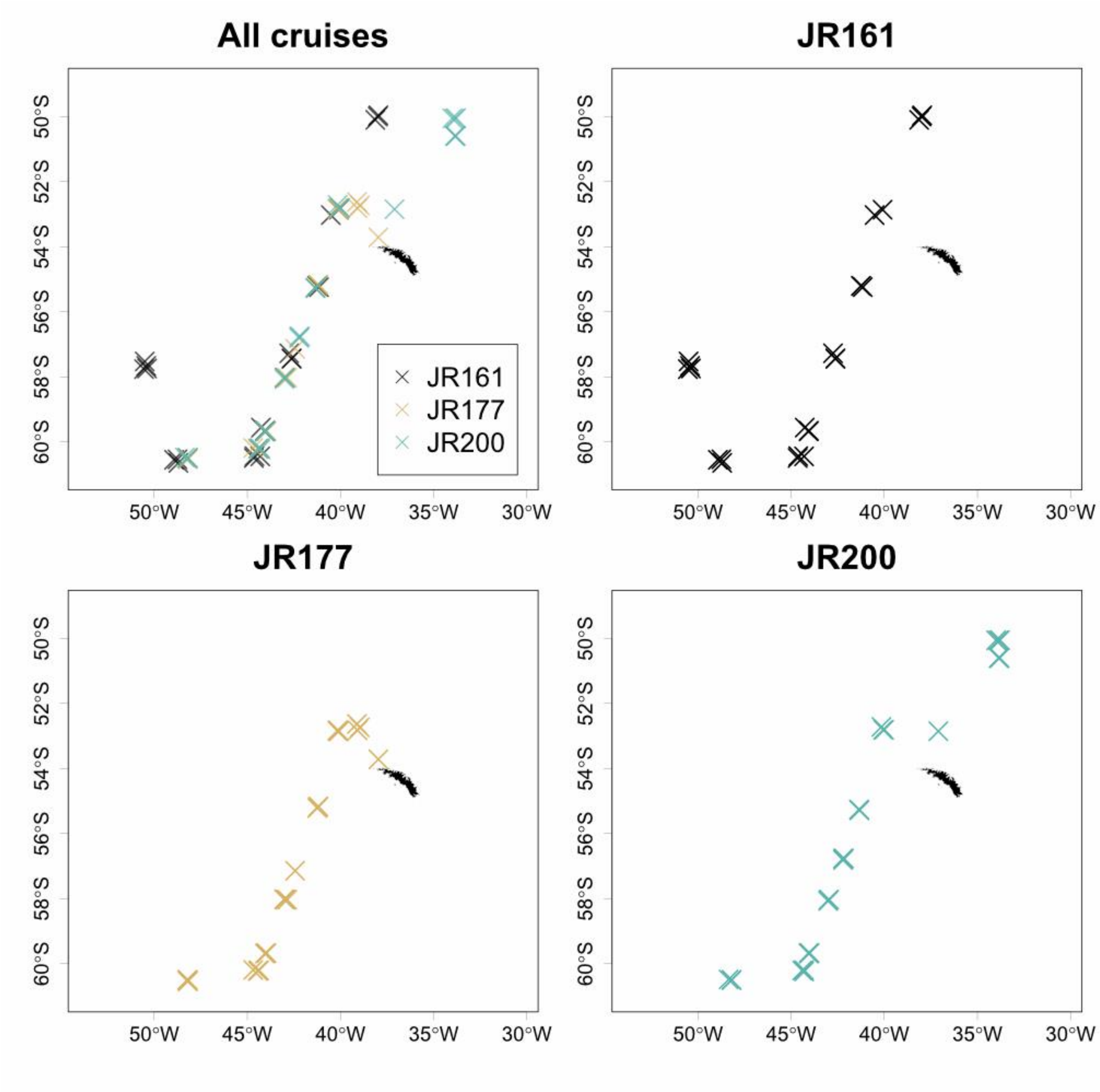

Figure S6: Distribution of zooplankton sampling stations from each cruise.

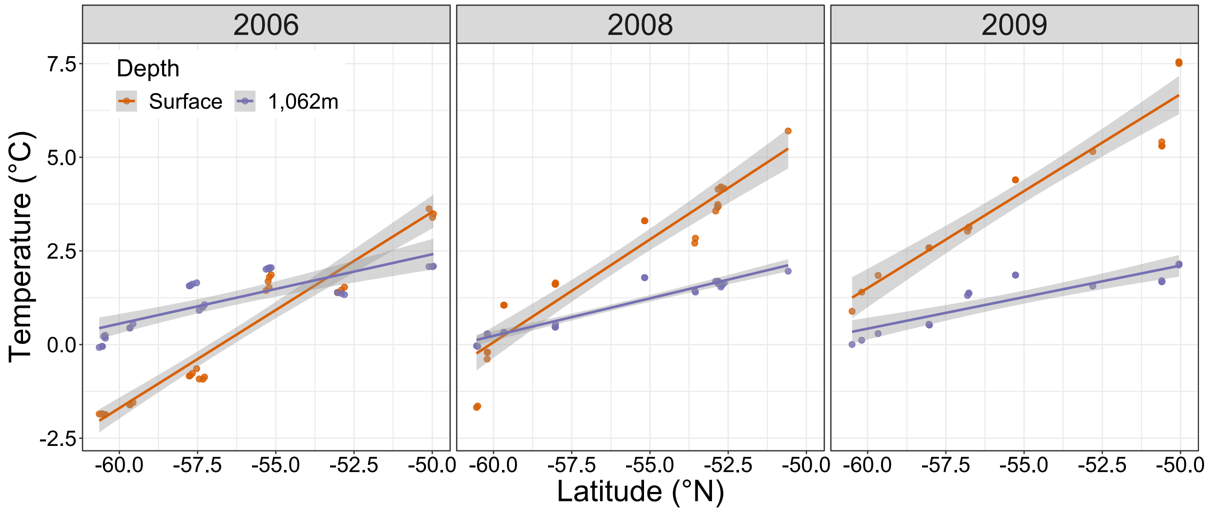

Figure S7: Comparison of temperatures at different depths across the sampling sites. Relationship between temperature (both at the surface and at 1,062m depth) and latitude at each haul location, split by sampling year (*n* = 27, 25, and 18 sites in 2006, 2008 and 2009, respectively). Lines represent model predicted values and shading represents 95% confidence intervals.

Supplementary Tables

Table S1: Identification of the optimal random effects and variance weighting structure for the models involving predator-prey mass ratio (PPMR). The table displays the various random effects and variance weighting structures for the linear mixed effects models describing the relationship between PPMR and the predictors sea-surface temperature (SST) and surface chlorophyll-a concentration (Chl-a), plus their interaction. The most parsimonious model structure based on Akaike's Information Criterion (AIC) is highlighted in grey. NC indicates models with no convergence.

Random effects structure						Variance structure								AIC
~1|Species	~1|Year	~SST|Species	~Chl-a|Species	~SST|Year	~Chl-a|Year	varIdent(~1|Species)	varIdent(~1|Year)	varFixed(~SST)	varFixed(~Chl-a)	varExp(~SST)	varExp(~Chl-a)	varConst(~SST)	varConst(~Chl-a)	
														3174.23
x														2920.04
	x													3167.52
x	x													2862.79
x		x												2923.84
x			x											2921.24
x		x	x											2926.99
	x			x										3156.83
	x				x									3158.10
	x			x	x									3156.01
x	x			x										NC
x	x				x									NC
x	x			x	x									NC
x	x	x												2852.11
x	x		x											2856.31
x	x	x	x											3000.85
x	x		x	x										NC
x	x		x		x									NC
x	x	x		x										NC
x	x	x			x									2856.11
x	x	x												2852.11
x	x	x				x								2746.44
x	x	x					x							2837.77
x	x	x				x	x							2734.96
x	x	x						x						3413.57
x	x	x							x					2823.82
x	x	x								x				2849.13
x	x	x									x			2825.19
x	x	x										x		NC
x	x	x											x	2815.18

Table S2: Identification of the optimal fixed effects structure for the models involving predator-prey mass ratio (PPMR). The table shows the various fixed effects structures for the linear mixed effects models describing the relationship between PPMR and the predictors sea-surface temperature (SST) and surface chlorophyl-a concentration (Chl-a). Each model includes the optimal random effects and variance weighting structure identified in Table S1. The most parsimonious model structure based on Akaike's Information Criterion (AIC) and retaining only significant fixed effects is highlighted in grey.

Fixed effects structure	AIC
SST*Chl-a	2719.88
SST+Chl-a	2718.18
Chl-a	2726.46
SST	2717.16
Null	2725.11

Table S3: Identification of the optimal random effects and variance weighting structure for the models involving predator body mass. The table shows the random effects and variance weighting structures for the linear mixed effects models describing the relationship between predator mass and the predictors sea-surface temperature (SST) and surface chlorophyll-a concentration (Chl-a), plus their interaction. The most parsimonious model structure based on Akaike's Information Criterion (AIC) is highlighted in grey.

Random effects structure						Variance structure								AIC
~1|Species	~1|Year	~SST|Species	~Chl-a|Species	~SST|Year	~Chl-a|Year	varIdent(~1|Species)	varIdent(~1|Year)	varFixed(~SST)	varFixed(~Chl-a)	varExp(~SST)	varExp(~Chl-a)	varConst(~SST)	varConst(~Chl-a)	
														1714.07
x														807.08
	x													1666.15
x	x													733.51
x		x												759.16
x			x											NC
x		x	x											762.26
	x			x										1662.24
	x				x									1651.06
	x			x	x									1656.18
x	x			x										737.41
x	x				x									737.51
x	x			x	x									743.41
x	x	x												709.89
x	x		x											713.01
x	x	x	x											1498.84
x	x		x	x										NC
x	x		x		x									716.87
x	x	x		x										713.89
x	x	x			x									713.88
x	x	x												709.89
x	x	x				x								167.11
x	x	x					x							650.81
x	x	x				x	x							126.01
x	x	x						x						864.08
x	x	x							x					729.57
x	x	x								x				693.14
x	x	x									x			706.43
x	x	x										x		NC
x	x	x											x	700.55

Table S4: Identification of the optimal fixed effects structure for the models involving predator mass. The table displays the fixed effects structures for the linear mixed effects models describing the relationship between predator mass and the predictors sea-surface temperature (SST) and surface chlorophyl-a concentration (Chl-a). Each model includes the optimal random effects and variance weighting structure identified in Table S3. The most parsimonious model structure based on Akaike's Information Criterion (AIC) and retaining only significant fixed effects is highlighted in grey.

Fixed effects structure	AIC
SST*Chl-a	105.79
SST+Chl-a	103.83
Chl-a	108.06
SST	102.39
Null	106.49

Table S5: Identification of the optimal random effects and variance weighting structure for the models involving dietary prey body mass. The table shows the random effects and variance weighting structures for linear mixed effects models describing the relationship between abundance-weighted prey mass in predator diets and the predictors sea-surface temperature (SST) and surface chlorophyll-a concentration (Chl-a), plus their interaction. The most parsimonious model structure based on Akaike's Information Criterion (AIC) is highlighted in grey. NC indicates models with no convergence.

Random effects structure						Variance structure								AIC
~1|Species	~1|Year	~SST|Species	~Chl-a|Species	~SST|Year	~Chl-a|Year	varIdent(~1|Species)	varIdent(~1|Year)	varFixed(~SST)	varFixed(~Chl-a)	varExp(~SST)	varExp(~Chl-a)	varConst(~SST)	varConst(~Chl-a)	
														3227.59
x														3059.97
	x													3208.49
x	x													2909.48
x		x												3051.49
x			x											NC
x		x	x											NC
	x			x										3181.53
	x				x									3186.04
	x			x	x									NC
x	x			x										NC
x	x				x									NC
x	x			x	x									NC
**x**	**x**	**x**												**2888.69**
x	x		x											2909.44
x	x	x	x											3055.41
x	x		x	x										NC
x	x		x		x									NC
x	x	x		x										NC
x	x	x			x									NC
x	x	x												2888.69
x	x	x				x								2744.95
x	x	x					x							2868.50
**x**	**x**	**x**				**x**	**x**							**2732.40**
x	x	x						x						3493.64
x	x	x							x					2861.30
x	x	x								x				2869.58
x	x	x									x			2861.37
x	x	x										x		2868.64
x	x	x											x	2851.97

Table S6: Identification of the optimal fixed effects structure for the models involving dietary prey body mass. The table displays the fixed effects structures for the linear mixed effects models describing the relationship between abundance-weighted prey mass in predator diets and the predictors sea-surface temperature (SST) and surface chlorophyl-a concentration (Chl-a). Each model includes the optimal random effects and variance weighting structure identified in Table S5. The most parsimonious model structure based on Akaike's Information Criterion (AIC) and excluding non-significant fixed effects is highlighted in grey.

Fixed effects structure	AIC
SST*Chl-a	2717.49
SST+Chl-a	2715.61
Chl-a	2714.59
SST	2713.76
Null	2712.70

Table S7: The results of a Moran's I test on the residuals from each selected linear mixed effects model with sea-surface temperature (SST).

Response	Moran's I	P value
Predator-prey mass ratio	0.003	0.339
Predator mass	-0.002	0.656
Prey mass in the diet	-0.001	0.061

Table S8: Identification of the optimal random effects and variance weighting structure for the models involving predator-prey mass ratio (PPMR) and temperature at 1,062 m depth. The table shows the random effects and variance weighting structures for the linear mixed effects models describing the relationship between PPMR and the predictors temperature at 1,062m depth (TAD) and surface chlorophyll-a concentration (Chl-a), plus their interaction. The most parsimonious model structure based on Akaike's Information Criterion (AIC) is highlighted in grey. NC indicates models with no convergence.

Random effects structure						Variance structure								AIC
Species~1	Year~1	Species~TAD	Species~Chl-a	Year~ TAD	Year~Chl-a	varIdent(~1|Species)	varIdent(~1|Year)	varFixed(~TAD)	varFixed(~Chl-a)	varExp(~TAD)	varExp(~Chl-a)	varConst(~TAD)	varConst(~Chl-a)	
														3178.22
x														2942.28
	x													3173.63
x	x													2883.36
x		x												2945.36
x			x											2943.27
x		x	x											2945.37
	x			x										3161.19
	x				x									3161.62
	x			x	x									3153.99
x	x			x										NC
x	x				x									NC
x	x			x	x									NC
x	x	x												2865.27 (highlighted grey)
x	x		x											2877.08
x	x	x	x											2994.12
x	x		x	x										NC
x	x		x		x									NC
x	x	x		x										NC
x	x	x			x									NC
x	x	x												2865.27
x	x	x				x								2762.27
x	x	x					x							2852.23
x	x	x				x	x							2751.60 (highlighted grey)
x	x	x						x						6959.02
x	x	x							x					2836.48
x	x	x								x				2811.59
x	x	x									x			2839.16
x	x	x										x		2833.85
x	x	x											x	2828.41

Table S9: Identification of the optimal fixed effects structure for the models involving predator-prey mass ratio (PPMR) and temperature at 1,062 m depth. The table displays the fixed effects structures for the linear mixed effects models describing the relationship between PPMR and the predictors temperature at 1,062m depth (TAD) and surface chlorophyl-a concentration (Chl-a). Each model includes the optimal random effects and variance weighting structure identified in Table S8. The most parsimonious model structure based on Akaike's Information Criterion (AIC) and retaining only significant fixed effects is highlighted in grey.

Fixed effects structure	AIC
TAD*Chl-a	2740.15
TAD+Chl-a	2738.21
Chl-a	2740.84
TAD	2739.64
Null	2741.57

Table S10: Identification of the optimal random effects and variance weighting structure for the models involving predator body mass and temperature at 1,062 m depth. The table shows the random effects and variance weighting structures for the linear mixed effects models describing the relationship between predator mass and the predictors temperature at 1,062m depth (TAD) and surface chlorophyl-a concentration (Chl-a), plus their interaction. The most parsimonious model structure based on Akaike's Information Criterion (AIC) is highlighted in grey. NC indicates models with no convergence.

Random effects structure						Variance structure								AIC
Species~1	Year~1	Species~TAD	Species~Chl-a	Year~ TAD	Year~Chl-a	varIdent(~1|Species)	varIdent(~1|Year)	varFixed(~TAD)	varFixed(~Chl-a)	varExp(~TAD)	varExp(~Chl-a)	varConst(~TAD)	varConst(~Chl-a)	
														1671.26
x														822.32
	x													1671.09
x	x													734.48
x		x												780.53
x			x											NC
x		x	x											780.29
	x			x										NC
	x				x									1659.41
	x			x	x									NC
x	x			x										NC
x	x				x									NC
x	x			x	x									NC
x	x	x												691.51
x	x		x											712.97
x	x	x	x											NC
x	x		x	x										NC
x	x		x		x									NC
x	x	x		x										NC
x	x	x			x									NC
x	x	x												691.51
x	x	x				x								155.92
x	x	x					x							636.45
x	x	x				x	x							113.34
x	x	x						x						3278.38
x	x	x							x					703.20
x	x	x								x				677.66
x	x	x									x			686.63
x	x	x										x		679.64
x	x	x											x	661.70

Table S11: Identification of the optimal fixed effects structure for the models involving predator mass and temperature at 1,062 m depth. The table displays the fixed effects structures for the linear mixed effects models describing the relationship between predator mass and the predictors temperature at 1,062m depth (TAD) and surface chlorophyl-a concentration (Chl-a). Each model includes the optimal random effects and variance weighting structure identified in Table S10. The most parsimonious model structure based on Akaike's Information Criterion (AIC) and retaining only significant fixed effects is highlighted in grey.

Fixed effects structure	AIC
TAD*Chl-a	96.84
TAD+Chl-a	95.57
Chl-a	100.62
TAD	95.07
Null	99.88

Table S12: Identification of the optimal random effects and variance weighting structure for the models involving dietary prey body mass and temperature at 1,062 m depth. The table shows the random effects and variance weighting structures for linear mixed effects models describing the relationship between abundance-weighted prey mass in predator diets and the predictors temperature at 1,062m depth (TAD) and surface chlorophyll-a concentration (Chl-a), plus their interaction. The most parsimonious model structure based on Akaike's Information Criterion (AIC) is highlighted in grey. NC indicates models with no convergence.

Random effects structure						Variance structure								AIC
Species~1	Year~1	Species~TAD	Species~Chl-a	Year~ TAD	Year~Chl-a	varIdent(~1|Species)	varIdent(~1|Year)	varFixed(~TAD)	varFixed(~Chl-a)	varExp(~TAD)	varExp(~Chl-a)	varConst(~TAD)	varConst(~Chl-a)	
														3199.70
x														3057.92
	x													3187.10
x	x													2912.83
x		x												3053.89
x			x											NC
x		x	x											NC
	x			x										3149.94
	x				x									3162.89
	x			x	x									3146.42
x	x			x										NC
x	x				x									NC
x	x			x	x									NC
x	x	x												2881.77
x	x		x											2913.04
x	x	x	x											NC
x	x		x	x										NC
x	x		x		x									NC
x	x	x		x										NC
x	x	x			x									2885.77
x	x	x												2881.77
x	x	x				x								2741.32
x	x	x					x							2861.62
x	x	x				x	x							2728.55
x	x	x						x						7247.49
x	x	x							x					2855.30
x	x	x								x				2782.81
x	x	x									x			2855.72
x	x	x										x		2818.68
x	x	x											x	2845.75

Table S13: Identification of the optimal fixed effects structure for the models involving dietary prey mass and temperature at 1,062 m depth. The table shows the fixed effects structures for the linear mixed effects models describing the relationship between abundance-weighted prey mass in predator diets and the predictors temperature at 1,062m depth (TAD) and surface chlorophyl-a concentration (Chl-a). Each model includes the optimal random effects and variance weighting structure identified in Table S12. The most parsimonious model structure based on Akaike's Information Criterion (AIC) and excluding non-significant fixed effects is highlighted in grey.

Fixed effects structure	AIC
TAD*Chl-a	2717.22
TAD +Chl-a	2715.43
Chl-a	2713.89
TAD	2713.79
Null	2712.36

Table S14: Model statistics for the effects of temperature at 1,062 m depth on predator and prey body masses. Output from linear mixed effects models with predator-prey mass ratio (PPMR), predator body mass and abundance-weighted average prey body mass in predator stomachs as response variables. The temperature variable represents temperature at ~1,062m depth (TAD), while R^2m and R^2c are the Nakagawa's marginal and conditional model R^2 values, respectively.

Model	Coefficient	Estimate	SE	DF	t-value	p-value
PPMR	Intercept	3.017	0.109	1550	27.717	<0.0001
	TAD	-0.131	0.050	1550	-2.596	0.0095
R^2m = 0.025, R^2c = 0.489						
Predator body mass	Intercept	0.566	0.108	1550	5.257	<0.0001
	TAD	-0.071	0.036	1550	-1.984	0.0475
R^2m = 0.012, R^2c = 0.980						
Mean prey body mass	Intercept	-2.357	0.072	1551	-32.600	<0.0001
R^2m < 0.001, R^2c = 0.476						

Table S15: The results of a Moran's I test on the residuals from each selected linear mixed effects model with the temperature at 1,062m depth as the predictor variable.

Response	Moran's I	p value
Predator-prey mass ratio	0.003	0.359
Predator mass	0.001	0.693
Prey mass in diet	0.003	0.312

Table S16: Identification of the optimal random effects and variance weighting structure for the models involving predator mass and a larger dataset of myctophid body sizes (*n* = 6,143). The table shows the random effects and variance weighting structures for linear mixed effects models describing the relationship between predator mass and the predictors sea-surface temperature (SST) and chlorophyll-a concentration (Chl-a), plus their interaction, using the larger dataset of myctophid body sizes. The most parsimonious model structure based on Akaike's Information Criterion (AIC) is highlighted in grey. NC indicates models with no convergence.

Random effects structure						Variance structure								AIC
Year~1	Species~1	Year~ SST	Species~SST	Year~ Chl-a	Species~Chl-a	varIdent(~1|Year)	varIdent(~1|Species)	varFixed(~SST)	varFixed(~Chl-a)	varExp(~SST)	varExp(~Chl-a)	varConst(~SST)	varConst(~Chl-a)	
														7542.835
x														7286.548
	x													4474.619
x	x													4396.677
x		x												7178.632
x				x										7203.550
x		x		x										NC
	x		x											4307.066
	x				x									NC
	x		x		x									4308.701
x	x	x												4349.700
x	x			x										7178.632
x	x	x		x										NC
x	x		x											4245.069
x	x				x									4282.719
x	x		x		x									6738.586
x	x	x	x											4249.069
x	x	x			x									4283.036
x	x		x	x										4183.526
x	x			x	x									NC
x	x		x	x										4183.526
						x								4157.655
							x							2161.584
						x	x							2158.628
								x						5708.168
									x					NC
										x				4129.790

	x			4184.524
		x		4106.948
			x	4186.680

Table S17: Identification of the optimal fixed effects structure for the models involving predator mass and a larger dataset of myctophid body sizes (*n* = 6,143). The table displays the fixed effects structures for the linear mixed effects model describing the relationship between predator size and the predictors sea-surface temperature (SST) and surface chlorophyll-a concentration (Chl-a), plus their interaction, using the larger dataset of myctophid body sizes. Each model includes the optimal random effects and variance weighting structure identified in Table S16. The most parsimonious model structure based on Akaike's Information Criterion (AIC) and retaining only significant fixed effects is highlighted in grey. The result of a Moran's I test for spatial autocorrelation for the optimal model is also provided.

Fixed effects structure	AIC	Moran's I	P-value
SST*Chl-a	2330.480		
SST+Chl-a	2328.769		
Chl-a	2426.262		
SST	2326.877	0.001	0.161
Null	2424.297		

Table S18: Model statistics for the optimal linear mixed effects model identified in Tables S16-S17 describing the relationship between sea-surface temperature (SST) and predator body mass using a larger dataset of myctophid body sizes (*n* = 6,143).

Coefficient	Estimate	SE	DF	t-value	p-value
Intercept	0.456	0.072	6113	6.351	<0.0001
SST	-0.026	0.002	6113	-10.397	<0.0001

Table S19: Identification of the optimal random effects and variance weighting structure for the models involving predator mass for each myctophid species. The table shows the random effects and variance weighting structures for linear mixed effects models describing the relationship between predator mass and sea-surface temperature (SST) for each species, using a larger dataset of myctophid body sizes (*n* = 6,143). The most parsimonious model based on Akaike's Information Criterion (AIC) is highlighted in grey. NC indicates models with no convergence.

Species	Random effect structure		Variance structure				AIC
	Year~1	Year~ sst	varident(~1|Year)	varfixed(~SST)	varexp(~SST)	varConst(~SST)	
							- 1288.456
	x						- 1287.623
	x	x					NC
E. carlsbergi			x				- 1318.993
				x			-1096.769
					x		-1309.503
						x	-1308.141
							1620.508
	x						1615.725
	x	x					1619.725
E. antarctica	x		x				1617.816
	x			x			2093.591
	x				x		1590.430
	x					x	1557.973
							51.531
	x						44.042
	x	x					NC
G. fraseri	x		x				42.658
	x			x			50.821
	x				x		45.530
	x					x	45.833
							119.246
	x						120.740
	x	x					NC
G. nicholsi			x				118.141
				x			94.101
					x		67.816
						x	90.010
G. braueri							1457.564

	x						1405.256
	x	x					1407.795
	x		x				1396.838
	x			x			1670.105
	x				x		1365.444
	x					x	1370.079
							942.875
	x						944.619
	x	x					NC
K. anderssoni			x				840.444
				x			915.442
					x		866.609
						x	880.390
							-47.476
	x						-45.476
	x	x					-41.476
N. achirus			x				-47.247
				x			-2.504
					x		-57.541
						x	-55.363
							-136.615
	x						-428.244
	x	x					NC
P. tenisoni			x				-443.422
				x			-427.005
					x		-443.606
						x	-459.302
							-44.103
	x						-55.174
	x	x					NC
P. bolini	x		x				-63.431
	x			x			-21.393
	x				x		-64.357
	x					x	-70.513
							-77.654
	x						-75.654
	x	x					NC
P. choriodon			x				-75.660
				x			-81.037
					x		-79.060
						x	NC

Table S20: Identification of the optimal fixed effects structure for the models involving predator mass for each myctophid species. The table shows the fixed effects structures for the linear mixed effects models of the relationship between predator size and sea-surface temperature (SST) for each species, using a larger dataset of myctophid body sizes (*n* = 6,143). Each model includes the optimal random effects and variance weighting structure identified in Table S19. The results of a Moran's I test for spatial autocorrelation are provided for the optimal models, along with the optimal correlation structure implemented based on AIC for any models with significant autocorrelation. No adjustments were made for multiple comparisons.

Species	Fixed effects structure	AIC	Moran's I	Moran's I p-value	Autocorrelation structure
E. carlsbergi	SST	-1340.507			
	Null	-1342.335	<0.001	0.7174	
E. antarctica	SST	1542.642	0.026	<0.0001	Rational
	Null	1569.967			
G. fraseri	SST	34.019	0.060	0.0038	Exponential
	Null	68.081			
G. nicholsi	SST	55.946	0.004	0.6894	
	Null	71.849			
G. braueri	SST	1356.068	0.009	0.0175	Exponential
	Null	1366.125			
K. anderssoni	SST	824.964	-0.010	0.0342	Spherical
	Null	1001.208			
N. achirus	SST	-72.145			
	Null	-73.502	-0.104	0.0914	
P. tenisoni	SST	-469.989	-0.004	0.9498	
	Null	-442.164			
P. bolini	SST	-83.925	-0.013	0.0937	
	Null	-76.457			
P. choriodon	SST	-93.411	-0.021	0.9246	
	Null	-78.834			

Table S21: Outputs of the optimal linear mixed effects models identified in Tables S19-S20, describing the relationship between predator mass and sea-surface temperature (SST) for each species, using a larger dataset of myctophid body sizes (*n* = 6,143).

Species	*Coefficient*	*Estimate*	*SE*	*DF*	*t-value*	*p-value*
E. carlsbergi	Intercept	0.790	0.003	486	298.685	<0.0001
E. antarctica	Intercept	0.579	0.021	2097	27.425	<0.0001
	SST	-0.022	0.009	2097	-2.479	0.0148
G. fraseri	Intercept	0.887	0.121	139	7.353	<0.0001
	SST	-0.100	0.028	139	-3.536	0.0011
G. nicholsi	Intercept	1.390	0.030	68	45.721	<0.0001
	SST	-0.083	0.019	68	-4.478	<0.0001
G. braueri	Intercept	0.754	0.079	1444	9.578	<0.0001
	SST	-0.083	0.007	1444	-11.213	<0.0001
K. anderssoni	Intercept	0.154	0.019	944	8.287	<0.0001
	SST	-0.085	0.006	944	-14.086	<0.0001
N. achirus	Intercept	0.153	0.015	76	9.965	<0.0001
P. tenisoni	Intercept	-0.294	0.153	213	-1.925	0.0555
	SST	0.030	0.005	213	6.484	<0.0001
P. bolini	Intercept	0.069	0.038	592	1.835	0.0670
	SST	-0.017	0.005	592	-3.406	0.0007
P. choriodon	Intercept	0.800	0.035	64	22.598	<0.0001
	SST	-0.050	0.009	64	-5.464	<0.0001

Table S22: Identification of the optimal random effects and variance weighting structure for the models involving myctophid species diversity. The table shows the random effects and variance weighting structures for the linear mixed effects models describing the relationship between myctophid species diversity (Shannon-Wiener index) and the interaction between sea-surface temperature (SST) and surface chlorophyll-a concentration (Chl-a). The most parsimonious model structure based on Akaike's Information Criterion (AIC) is highlighted in grey. NC indicates models with no convergence.

Random effects structure			Variance structure							AIC
Year~1	Year~ SST	Year~ Chl-a	varIdent(~1|Year)	varFixed(~SST)	varFixed(~Chl-a)	varExp(~SST)	varExp(~Chl-a)	varConst(~SST)	varConst(~Chl-a)	
										134.843
x										136.498
x	x									137.938
x		x								139.111
x	x	x								NC
			x							131.819
				x						169.6902
					x					155.0526
						x				132.8421
							x			132.0601
								x		137.1728
									x	129.3736

Table S23: Identification of the optimal fixed effects structure for the models involving myctophid species diversity. The table displays the fixed effects structures for the linear mixed effects models of the relationship between Shannon-Wiener diversity and the interaction between sea-surface temperature (SST) and surface chlorophyll-a concentration (Chl-a). Each model includes the optimal random effects and variance weighting structure identified in Table S22. The most parsimonious model structure based on Akaike's Information Criterion (AIC) and retaining only significant fixed effects is highlighted in grey. The results of a Moran's I test for spatial autocorrelation is provided for the optimal model.

Fixed effects structure	AIC	Moran's I	P-value
SST*Chl-a	114.7948		
SST+Chl-a	115.1217		
Chl-a	148.1829		
SST	114.1337	0.071	0.103
Null	146.3198		

Table S24: Model statistics for the optimal linear mixed effects model identified in Tables S22-S23 describing the relationship between myctophid species diversity and sea-surface temperature (SST).

Coefficient	Estimate	SE	DF	t-value	p-value
Intercept	0.962	0.045	115	21.545	<0.0001
SST	0.090	0.015	115	6.080	<0.0001

Table S25: Length-Mass relationships used to estimate body mass of each individual myctophid species. The regressions used to convert standard length (SL, mm) to wet mass (WM, g) follow the equation WM = a * SL^b. Lower and upper 95% confidence intervals are provided for each coefficient, along with the overall R^2 for the relationship.

Species	*a*	*Lower*	*Upper*	*b*	Lower	Upper	R^2
E. carlsbergi	2.09 x10^{-05}	9.51 x10^{-06}	4.59 x10^{-05}	2.90	2.72	3.08	0.7214
E. antarctica	3.72 x10^{-06}	3.22 x10^{-06}	4.30X10^{-06}	3.27	3.24	3.31	0.9599
G. fraseri	3.53 x10^{-06}	1.31 x10^{-06}	9.51 x10^{-06}	3.24	3.00	3.47	0.8811
G. nicholsi	2.87 x10^{-06}	2.02 x10^{-06}	4.08 x10^{-06}	3.25	3.18	3.33	0.9936
G. braueri	4.58 x10^{-06}	3.60 x10^{-06}	5.82 x10^{-06}	3.11	3.06	3.17	0.9326
K. anderssoni	9.05 x10^{-06}	7.49 x10^{-06}	1.09 x10^{-05}	3.02	2.97	3.07	0.9599
N. achirus	8.14 x10^{-06}	5.17x 10^{-07}	1.28 x10^{-02}	2.49	1.45	3.54	0.4259
P. tenisoni	1.39 x10^{-05}	9.74x 10^{-06}	1.97 x10^{-05}	2.94	2.84	3.03	0.9589
P. bolini	1.98 x10^{-05}	1.34 x10^{-05}	2.92 x10^{-05}	2.88	2.77	2.98	0.8926
P. choriodon	1.27 x10^{-05}	3.24 x10^{-06}	4.94 x10^{-05}	2.98	2.66	3.30	0.8779

Table S26: Number of stomachs collected for each myctophid species during each cruise.

Species	JR161	JR177	JR200	TOTAL
Electrona carlsbergi	80	34	27	141
Electrona antarctica	152	178	112	442
Gymnoscopelus fraseri	11	60	27	98
Gymnoscopelus nicholsi	22	11	7	40
Gymnoscopelus braueri	143	94	109	346
Krefftichthys anderssoni	132	44	22	198
Nannobranchium achirus	23	0	0	23
Protomyctophum tenisoni	27	17	0	44
Protomyctophum bolini	106	76	26	208
Protomyctophum choriodon	0	36	0	36
TOTAL	696	550	330	1576

Table S27: Number of individual body mass measurements for each myctophid species during each cruise, from a larger dataset of myctophid sizes (from which only a subset were kept for stomach contents analyses).

Species	JR161	JR177	JR200	TOTAL
E. carlsbergi	195	248	43	486
E. antarctica	568	1023	510	2101
G. fraseri	12	90	41	143
G. nicholsi	30	30	8	68
G. braueri	443	576	429	1448
K. anderssoni	590	162	192	944
N. achirus	43	9	24	76
P. tenisoni	79	98	40	217
P. bolini	261	177	158	596
P. choriodon	0	50	14	64
TOTAL	2221	2463	1459	6143

Table S28: Mean abundances for each broad zooplankton taxon, averaged across sampling sites.

Taxon	Mean density (ind./m^2)	Proportion of total density
Copepoda	13,822.938	0.743
Polychaeta & Chaetognatha	2,645.003	0.142
Pteropoda	1,076.652	0.058
Ostracoda	780.023	0.042
Euphausidae	109.152	0.006
Cnidaria	107.666	0.006
Tunicata	41.255	0.002
Amphipoda	7.266	<0.001
Decapoda	3.692	<0.001
Cephalopoda	0.013	<0.001
Isopoda	0.003	<0.001
Mysidae	0.001	<0.001

Table S29: Identification of the optimal random effects and variance weighting structure for the models involving predator dietary prey size selectivity. The table shows the random effects and variance weighting structures for linear mixed effects models describing the relationship between predator dietary size preference and the interaction between sea-surface temperature (SST) and predator body mass. The most parsimonious model structure based on Akaike's Information Criterion (AIC) is highlighted in grey. NC indicates models with no convergence.

Random effect structure					Variance structure					AIC
~1|Species	~1|Site	~1|Year	~SST|Species	~SST|Year	varIdent(~1|Species)	varIdent(~1|Year)	varFixed(~SST)	varExp(~SST)	varConst(~SST)	
										288.76
x										263.06
	x									268.13
		x								275.76
x	x									243.02
x		x								238.30
	x	x								266.08
x	x	x								243.80
x			x							264.69
x				x						269.55
	x		x							NC
	x			x						274.13
x	x	x	x							244.75
x	x	x		x						NC
x	x	x	x	x						NC
x		x								238.30
x		x			x					213.95
x		x				x				216.12
x		x			x	x				208.60
x		x					x			226.08
x		x						x		231.49
x		x							x	227.54

Table S30: Identification of the optimal fixed effects structure for the models involving dietary prey size selectivity. The table displays the fixed effects structures for the linear mixed effects models describing the relationship between predator dietary size preference and the interaction between sea-surface temperature (SST) and predator body mass. Each model includes the optimal random effects and variance weighting structure identified in Table S29. The most parsimonious model structure based on Akaike's Information Criterion (AIC) and retaining only significant fixed effects is highlighted in grey.

Fixed effects structure	AIC	Moran's I	P value
SST*predator mass	192.34	-0.025	0.515
SST+predator mass	200.35		
Predator mass	201.51		
SST	232.45		
Null	236.86		
