# Supplementary material for: Temperature alters the predator-prey size relationships and size-selectivity of Southern Ocean fish
Source: Nat Commun. 2024 May 10;15:3979. doi: 10.1038/s41467-024-48279-0 (PMC11087476; doi:10.1038/s41467-024-48279-0)
Supplement: Supplementary file 3 — Reporting Summary [file 41467_2024_48279_MOESM3_ESM.pdf]

nature portfolio

Corresponding author(s): Patrick Eskuche-Keith

Last updated by author(s): Apr 4, 2024

Reporting Summary

Nature Portfolio wishes to improve the reproducibility of the work that we publish. This form provides structure for consistency and transparency in reporting. For further information on Nature Portfolio policies, see our Editorial Policies and the Editorial Policy Checklist.

Statistics

For all statistical analyses, confirm that the following items are present in the figure legend, table legend, main text, or Methods section.

n/a	Confirmed	
☐	☒	The exact sample size (*n*) for each experimental group/condition, given as a discrete number and unit of measurement
☐	☒	A statement on whether measurements were taken from distinct samples or whether the same sample was measured repeatedly
☐	☒	The statistical test(s) used AND whether they are one- or two-sided<br>*Only common tests should be described solely by name; describe more complex techniques in the Methods section.*
☐	☒	A description of all covariates tested
☐	☒	A description of any assumptions or corrections, such as tests of normality and adjustment for multiple comparisons
☐	☒	A full description of the statistical parameters including central tendency (e.g. means) or other basic estimates (e.g. regression coefficient) AND variation (e.g. standard deviation) or associated estimates of uncertainty (e.g. confidence intervals)
☐	☒	For null hypothesis testing, the test statistic (e.g. *F*, *t*, *r*) with confidence intervals, effect sizes, degrees of freedom and *P* value noted<br>*Give P values as exact values whenever suitable.*
☒	☐	For Bayesian analysis, information on the choice of priors and Markov chain Monte Carlo settings
☒	☐	For hierarchical and complex designs, identification of the appropriate level for tests and full reporting of outcomes
☒	☐	Estimates of effect sizes (e.g. Cohen's *d*, Pearson's *r*), indicating how they were calculated

Our web collection on statistics for biologists contains articles on many of the points above.

Software and code

Policy information about availability of computer code

Data collection	No software was used for data collection
Data analysis	All analyses were conducted in R software version 4.3.0. R packages used: tidyverse 2.0.0; openxlsx 4.2.5.2; ncdf4 1.21; raster 3.6-20; rgdal 1.6-6; ggplot2 3.5.0; nlme 3.1-162; MuMIn 1.47.5; RColorBrewer 1.1-3; dplyr 1.2.2.; effects 4.2-2; ape 5.7-1; sp 1.6-0; gstat 2.1-1; ggridges 0.5.4. Maps were produced using QGIS 3.28 Firenze.

For manuscripts utilizing custom algorithms or software that are central to the research but not yet described in published literature, software must be made available to editors and reviewers. We strongly encourage code deposition in a community repository (e.g. GitHub). See the Nature Portfolio guidelines for submitting code & software for further information.

Data

Policy information about availability of data

All manuscripts must include a data availability statement. This statement should provide the following information, where applicable:

- Accession codes, unique identifiers, or web links for publicly available datasets
- A description of any restrictions on data availability
- For clinical datasets or third party data, please ensure that the statement adheres to our policy

The processed data have been deposited in the Zenodo database [https://doi.org/10.5281/zenodo.10879865]. The original raw fish stomach contents and zooplankton data used in this study are available in the UK Polar Data Centre [https://doi.org/10.5285/1f70d2a9-b0d7-4403-bcb9-2cd884222a9c; https://doi.org/10.5285/5798742d-dd5f-480b-8298-2c2b449cbab3; https://doi.org/10.5285/5a711904-ef42-46a3-9f47-3f0d6b231f65]. The SST and surface chlorophyll-a

data used in this study are available in the Copernicus Marine Service database [https://doi.org/10.48670/moi-0002 and https://doi.org/10.48670/moi-00278, respectively]. Source data are provided with this paper.

Research involving human participants, their data, or biological material

Policy information about studies with human participants or human data. See also policy information about sex, gender (identity/presentation), and sexual orientation and race, ethnicity and racism.

Reporting on sex and gender	N/A
Reporting on race, ethnicity, or other socially relevant groupings	N/A
Population characteristics	N/A
Recruitment	N/A
Ethics oversight	N/A

Note that full information on the approval of the study protocol must also be provided in the manuscript.

Field-specific reporting

Please select the one below that is the best fit for your research. If you are not sure, read the appropriate sections before making your selection.

☐ Life sciences ☐ Behavioural & social sciences ☒ Ecological, evolutionary & environmental sciences

For a reference copy of the document with all sections, see nature.com/documents/nr-reporting-summary-flat.pdf

Ecological, evolutionary & environmental sciences study design

All studies must disclose on these points even when the disclosure is negative.

Study description	This study uses samples of mesopelagic fish (family Myctophidae) and zooplankton collected at multiple stations across the Scotia Sea, Southern Ocean, during cruises in 2006, 2008 and 2009 to investigate changes in predator and prey body size relationships with temperature. A total of 6143 individual fish were collected from 67 hauls using a 25 m2 rectangular mid-water trawl net (RMT25), deployed at depth ranges of 0-200, 200-400, 400-700, and 700-1000 m. The dataset also includes the stomach contents for 1576 of these fish representing a further dataset of 3,707 prey records. The overall abundance of each myctophid species at each haul location is also available. A second dataset, also obtained from the UK Polar Data Centre, details the meso- and macro-zooplankton composition and densities in 46 hauls sampled from the same cruises using both RMT25 nets and paired bongo nets (mesh size 50µm).<br><br>Data were analysed using nested mixed effects models to take into account the year of sampling, sampling location and individual fish identity or prey taxon as random effects, depending on the specific analysis. The primary predictor in models was average sea surface temperature, obtained for each sampling station from a 1/12° gridded Copernicus Global Ocean Physics Reanalysis product (GLORYS12V1), with a minimum temperature of -1.87°C at a latitude of 60.45°S and a maximum temperature of 7.55°C at 50.05°S. Surface chlorophyll-a concentrations, obtained from the Copernicus-GlobColour dataset at a resoution of 4x4km, were also included as a predictor in models but were dropped during model selection due to non-significance.
Research sample	The data represent the mesopelagic lanternfish (family Myctophidae) community sampled opportunistically at 67 locations, obtained from the UK Polar Data Centre. Sampling locations were chosen to cover a broad range of environments to facilitate the variety of ecological, oceanographic and bio-geochemical projects undertaken by the British Antarctic Survey. These data were chosen as they provide the most comprehensive available overview of the species/size composition and diets of this highly abundant group of fish which play a central role in Southern Ocean food webs. A second dataset, also obtained from the UK Polar Data Centre, details the meso- and macro-zooplankton composition and densities in 46 hauls sampled from the same cruises. These data were selected as they represent the best available information on the community size structure of zooplankton in this region. The ages/sexes of organisms were not considered for this study.
Sampling strategy	Samples were collected at stations across a latitudinal gradient within the Scotia Sea, Southern Ocean. Overall sample size was not predetermined as data collection was opportunistic, though a random subsample of up to 25 fish per species was set aside for stomach dissection at each location
Data collection	The data used in this study were obtained from the UK Polar Data Centre (https://doi.org/10.5285/1f70d2a9-b0d7-4403-bcb9-2cd884222a9c; https://doi.org/10.5285/5798742d-dd5f-480b-8298-2c2b449cbab3 ; https://doi.org/10.5285/5a711904-ef42-46a3-9f47-3f0d6b231f65). Scientific personnel on board varied between cruises and can be found in the relevant cruise reports on the British Oceanographic Data Centre website (JR161, JR177, JR200). Fish were collected using a 25 m2 rectangular mid-water trawl net (RMT25), deployed at depth ranges of 0-200, 200-400, 400-700, and 700-1000 m. Fish were identified to the lowest possible taxonomic level and measured to the nearest millimeter standard length. A random subsample of up to 25 individuals per species were retained at each haul and frozen for later stomach contents analysis, where prey were identified, weighed and counted. Zooplankton samples were stored in 4% formalin and later identified, enumerated and weighed to estimate abundances and biomass.

Timing and spatial scale	Dates for each cruise were as follows: JR161 - Oct 24th to Dec 3rd 2006; JR177 - Dec 31st 2007 to Fev 16th 2008; JR200 - Mar 11th to Apr 18th 2009. These periods were selected as they cover the Spring, Summer and Autumn periods, respectively, which were of interest for various scientific projects at the time. Samples were taken between 50.05 and 60.45°S, covering the entire latitudinal range from South Georgia to the South Orkney Islands.
Data exclusions	A total of 8 individual fish were excluded from analyses of predator-prey body size relationships because they came from myctophid species that occurred extremely rarely (7 individuals of Gymnoscopelus opisthopterus, and 1 individual of G. piabilis) and were therefore not considered representative of their wider populations.
Reproducibility	The data were obtained opportunistically over the course of three costly and multi-disciplinary scientific cruises to a remote region. As such, it has not been possible to collect comparable data to test reproducibility.
Randomization	Non-independence of records was accounted for in our mixed effects modelling structure ('lme' function in the 'nlme' package of R) by including year, sampling location and individual fish or prey taxon as nested random effects, depending on the specific model being implemented. Additionally, the presence of spatial autocorrelation was tested for using Moran's I test, and spatial correlation structures were included in any models which had significant autocorrelation.
Blinding	This was not applicable to this study, as we were not conducting tests on multiple groups

Did the study involve field work? ☒ Yes ☐ No

Field work, collection and transport

Field conditions	Cruise narratives detailing daily conditions can be obtained from the following links: JR161 (https://www.bodc.ac.uk/resources/inventories/cruise_inventory/report/8262/); JR177 (https://www.bodc.ac.uk/resources/inventories/cruise_inventory/report/8276/); JR200 (https://www.bodc.ac.uk/resources/inventories/cruise_inventory/report/9357/). The sampling region covers multiple bioregions including a seasonal sea-ice zone at the southern extreme and ice-free open water at the northern extreme. There is a strong temperature gradient from around 0 degrees C at the most southerly point to almost 8 degrees C at the northern limit around the Antarctic Polar Front. Numerous frontal zones also generate localised productivity gradients, but these frontal zones are not believed to pose a significant barrier to the movement of zooplankton (https://www.sciencedirect.com/science/article/pii/S0967064511001950#s0090).
Location	Scotia Sea, Southern Ocean, between approximately 50-60 degrees South, and 30-50 degrees West. Water depths range from close to zero in shelf regions such as South Georgia to 6000m+ in the central Scotia Sea.
Access & import/export	As required by the Antarctic Treaty (www.ats.aq), all research was conducted after a preliminary environmental assessment and under permits issued by the UK Foreign and Commonwealth Office and the Government of South Georgia and South Sandwich Islands. Fishes and zooplankton were collected as part of faunal surveys and no animal experiments were conducted. Therefore, no ethical approval was required.
Disturbance	The primary source of disturbance during sampling was the use of pelagic RMT-25 trawls and bongo nets to sample fish and zooplankton. These disturbances were restricted in spatial and temporal extent and any disturbance was therefore extremely localised. As such, any negative impacts on biota and habitats were minimal. Sampling adhered to all environmental audits and requirements required as part of the relevant governing bodies/treaties.

Reporting for specific materials, systems and methods

We require information from authors about some types of materials, experimental systems and methods used in many studies. Here, indicate whether each material, system or method listed is relevant to your study. If you are not sure if a list item applies to your research, read the appropriate section before selecting a response.

Materials & experimental systems

n/a	Involved in the study	
☒	☐	Antibodies
☒	☐	Eukaryotic cell lines
☒	☐	Palaeontology and archaeology
☐	☒	Animals and other organisms
☒	☐	Clinical data
☒	☐	Dual use research of concern
☒	☐	Plants

Methods

n/a	Involved in the study	
☒	☐	ChIP-seq
☒	☐	Flow cytometry
☒	☐	MRI-based neuroimaging

Animals and other research organisms

Policy information about studies involving animals; ARRIVE guidelines recommended for reporting animal research, and Sex and Gender in Research

Laboratory animals	No laboratory animals were used
Wild animals	Fish were caught using an RMT25 net with a cod end mesh size of 5mm. Zooplankton were collected using either the RMT25 net or paired Bongo nets with 50 µm mesh size. Fish were processed and released as quickly as possible to maximise survival chances. The exception was the 25 individuals from each species retained for stomach contents dissection. These were dispatched humanely by brain spiking. Zooplankton samples were preserved in 4% formalin with seawater for later identification.
Reporting on sex	Sex of the fish or zooplankton was not considered relevant to the hypotheses investigated in this study.
Field-collected samples	No laboratory work involving live specimens was conducted
Ethics oversight	The data were collected following standard protocols and ethic approval from the British Antarctic Survey and the Environmental Protocol (1991) of the Antarctic Treaty

Note that full information on the approval of the study protocol must also be provided in the manuscript.

Plants

Seed stocks	N/A
Novel plant genotypes	N/A
Authentication	N/A
